# Neighborhood Socioeconomic Disadvantage Across the Life Course and Premature Mortality

**DOI:** 10.1001/jamanetworkopen.2024.26243

**Published:** 2024-08-01

**Authors:** Wayne R. Lawrence, Anna M. Kucharska-Newton, Jared W. Magnani, LaPrincess C. Brewer, Meredith S. Shiels, Kristen M. George, Pamela L. Lutsey, Brittany D. Jenkins, Kevin J. Sullivan, April P. Carson, Neal D. Freedman

**Affiliations:** Division of Cancer Epidemiology and Genetics, National Cancer Institute, National Institutes of Health, Rockville, Maryland (Lawrence, Shiels, Freedman); Department of Epidemiology, Gillings School of Global Public Health, University of North Carolina at Chapel Hill (Kucharska-Newton); Department of Medicine, University of Pittsburgh School of Medicine, Pittsburgh, Pennsylvania (Magnani); Heart and Vascular Institute, University of Pittsburgh Medical Center, Pittsburgh, Pennsylvania (Magnani); Department of Epidemiology, Graduate School of Public Health, University of Pittsburgh, Pittsburgh, Pennsylvania (Magnani); Department of Cardiovascular Medicine, Mayo Clinic College of Medicine, Rochester, Minnesota (Brewer); Center for Health Equity and Community Engagement Research, Mayo Clinic, Rochester, Minnesota (Brewer); Department of Public Health Sciences, University of California Davis School of Medicine (George); Division of Epidemiology and Community Health, University of Minnesota, Minneapolis (Lutsey); Department of Biochemistry and Molecular Biology, Johns Hopkins University, Baltimore, Maryland (Jenkins); Department of Medicine, University of Mississippi Medical Center, Jackson (Sullivan, Carson); Division of Cancer Control and Population Sciences, National Cancer Institute, National Institutes of Health, Rockville, Maryland (Freedman).

## Abstract

**IMPORTANCE:**

There are consistent data demonstrating that socioeconomic disadvantage is associated with risk of premature mortality, but research on the relationship between neighborhood socioeconomic factors and premature mortality is limited. Most studies evaluating the association between neighborhood socioeconomic status (SES) and mortality have used a single assessment of SES during middle to older adulthood, thereby not considering the contribution of early life neighborhood SES.

**OBJECTIVE:**

To investigate the association of life course neighborhood SES and premature mortality.

**DESIGN, SETTING, AND PARTICIPANTS:**

This cohort study included Black and White participants of the multicenter Atherosclerosis Risk in Communities Study, a multicenter study conducted in 4 US communities: Washington County, Maryland; Forsyth County, North Carolina; Jackson, Mississippi; and the northwestern suburbs of Minneapolis, Minnesota. Participants were followed up for a mean (SD) of 18.8 (5.7) years (1996–2020). Statistical analysis was performed from March 2023 through May 2024.

**EXPOSURE:**

Participants’ residential addresses during childhood, young adulthood, and middle adulthood were linked with US Census–based socioeconomic indicators to create summary neighborhood SES scores for each of these life epochs. Neighborhood SES scores were categorized into distribution-based tertiles.

**MAIN OUTCOMES AND MEASURES:**

Premature death was defined as all-cause mortality occurring before age 75 years. Multivariable-adjusted Cox proportional hazards models were used to estimate hazard ratios (HRs) and 95% CIs.

**RESULTS:**

Among 12 610 study participants, the mean (SD) age at baseline was 62.6 (5.6) years; 3181 (25.2%) were Black and 9429 (74.8%) were White; and 7222 (57.3%) were women. The lowest, compared with the highest tertile, of neighborhood SES score in middle adulthood was associated with higher risk of premature mortality (HR, 1.28; 95% CI, 1.07–1.54). Similar associations were observed for neighborhood SES in young adulthood among women (HR, 1.25; 95% CI, 1.00–1.56) and neighborhood SES in childhood among White participants (HR, 1.25; 95% CI, 1.01–1.56). Participants whose neighborhood SES remained low from young to middle adulthood had an increased premature mortality risk compared with those whose neighborhood SES remained high (HR, 1.25; 95% CI, 1.05–1.49).

**CONCLUSIONS AND RELEVANCE:**

In this study, low neighborhood SES was associated with premature mortality. The risk of premature mortality was greatest among individuals experiencing persistently low neighborhood SES from young to middle adulthood. Place-based interventions that target neighborhood social determinants of health should be designed from a life course perspective that accounts for early-life socioeconomic inequality.

## Introduction

The social and economic attributes of the neighborhood environment can affect health and quality of life by influencing access to resources and opportunities.^1–3^ The socioeconomic circumstances in which people live, from early childhood to older adulthood, contribute to health disparities.^1,3,4^ For instance, studies have documented that low neighborhood socioeconomic status (SES), compared with high neighborhood SES, is associated with greater chronic disease burden due to differences in access and quality of resources, such as employment, housing, and health care.^1,4,5^ Additionally, individuals who reside in socioeconomically disadvantaged neighborhoods are more likely to have limited access to fresh foods, be exposed to greater environmental toxins, and have fewer areas to safely engage in physical activity, all of which may contribute to health and lifespan.^3^

In the United States, premature all-cause mortality has increased in recent years, disproportionally among those with socioeconomic disadvantage.^6–8^ Although many studies have linked low SES to elevated risk of premature mortality, understanding the independent contribution of the neighborhood socioeconomic environment remains limited.^6,9–11^ Most epidemiologic studies have relied on measuring the neighborhood environment at 1 time point, most often in mid- to late life, thereby not investigating early life exposures or potential changes in neighborhood SES across age periods (life epochs).^3,12^ Also, the accumulation of exposure to adverse neighborhood conditions may affect disease onset and mortality.^3,13^ A life course approach provides a more comprehensive understanding of the contribution of early life factors and their relationship with later life health.^14^

In this investigation, we used life course measures from the Atherosclerosis Risk in Communities (ARIC) study to examine the association of neighborhood SES in childhood, young adulthood, and middle adulthood, as well as changes across adulthood life epochs, with premature mortality. We hypothesized that lower neighborhood SES at each life epoch and persistently low neighborhood SES across adulthood would each be associated with the risk of premature mortality.

## Methods

The ARIC Study is a large multicenter prospective cohort study investigating the etiology and natural history of atherosclerosis and cardiovascular disease. Details describing the ARIC Study cohort have been published elsewhere.^15^ Briefly, participants were recruited into the study through stratified random sampling from 4 US communities: Washington County, Maryland; Forsyth County, North Carolina; Jackson, Mississippi; and the northwestern suburbs of Minneapolis, Minnesota. A total of 15 792 participants aged 45 to 64 years at visit 1 in 1987 to 1989 were contacted annually to complete surveys on general health and hospitalizations. Multiple in-person clinic visits were conducted from baseline through the writing of this article (eFigure 1 in Supplement 1). Between 2001 and 2002, 12 716 participants provided their historical residential address information and completed questionnaires that evaluated their SES at different life epochs as part of the Life Course Socioeconomic, Social Context, and Cardiovascular Disease (LCSES) study, an ARIC ancillary study.^16^ Visit 4 (1996–1998), which aligned most closely in time with the LCSES study, was used as the study baseline for the current analysis. This study followed the Strengthening the Reporting of Observational Studies in Epidemiology (STROBE) reporting guidelines.^17^

We excluded participants with no information at visit 4 (n = 32) and those self-reporting as neither Black nor White race due to small sample size (n = 35). Black participants from Washington County, Maryland, and Minneapolis, Minnesota, field centers were excluded due to small numbers (n = 39). All participants provided informed consent, and institutional review board approval was obtained at each study site. The final analytic sample comprised 12 610 participants.

### Life Course SES

As part of the LCSES study, participants reported their addresses for the following age periods: at approximately age 10 years (childhood), 30 years (young adulthood), and 40 to 50 years (middle adulthood). Addresses collected were geocoded and linked to US Census data. Childhood neighborhood SES was based on Census information (1930–1950) at the county level, as this was the smallest geographic unit with aggregated data for all participating areas during this period. Young and middle adulthood neighborhood SES was evaluated on the basis of the most proximate US Census (1960–1980) at the census tract level. Previous validation studies have reported high repeatability and accuracy of geocodes in the ARIC study.^18,19^

Analytical details for the derivation of neighborhood SES measures at different life epochs have been previously described.^16,20,21^ Briefly, for every life epoch (childhood, young and middle adulthood), *z* scores were created for Census variables representing neighborhood-level educational attainment, home ownership, income, occupation, and property value and summed to create a composite neighborhood *z* score.^16,22^ A cumulative adulthood z score was created by summing the scores in young and middle adulthood. Higher *z* scores indicated greater neighborhood SES. Distribution-based tertiles were created for the adulthood cumulative neighborhood SES score and for each life epoch neighborhood SES score. We did not include childhood for cumulative neighborhood SES since that life epoch derived from county-level information, which cannot be compared with subsequent census tract–level data.

We also derived variables to evaluate changes in neighborhood SES between adulthood life epochs. This was performed by dichotomizing each life epoch at the median *z* score, then evaluating whether participants were stable or changed in reference to the median. The neighborhood SES patterns between young and middle adulthood included 4 categories: (1) low-to-high or improvement (below the median in young adulthood and at or above the median in middle adulthood), (3) high-to-low (decline), (3) low-to-low (stable-low), and (4) high-to-high (stable-high). This approach was used to estimate the neighborhood SES patterns from young to middle adulthood.

Individual socioeconomic position (SEP) in childhood, young adulthood, and middle adulthood were obtained via telephone questionnaires. Socioeconomic factors evaluated included educational attainment, occupation, occupational role, home ownership, and family income (eTable 1 in Supplement 1). The individual SEP scores were created by summarizing variables at the 3 life epochs as previously described.^16,23^ Response variables related to each life epoch values ranged between 0 (lowest) and 5 (highest). Scores were summed at each life epoch and categorized into tertiles based on population distribution, where higher scores represented higher individual SEP.

### Mortality

Follow-up time was defined as time (in days) between study baseline visit and mortality event or administrative censoring (December 31, 2020), whichever occurred first. In the event of study participant death, ARIC interviewers contacted participant proxy, obituaries, hospital records, death certificates, or vital statistics from the National Death Index.^15^ Premature death was defined as death from any cause occurring before age 75 years, which was the approximate average age of death among US adults throughout the study period.^24^

### Covariates

At ARIC visit 4, information obtained for descriptive assessment of study population characteristics included participants’ self-reported age, sex, race, education, household income, marital status, alcohol consumption, cigarette smoking, and physical activity.^15,22^ Additionally, participants’ clinical characteristics examined included body mass index (calculated as weight in kilograms divided by height in meters squared), hypertension status (diastolic blood pressure of >90 mm Hg, systolic blood pressure of >140 mm Hg, or self-reported antihypertensive medication use), and diabetes status (fasting glucose ≥126 mg/dL, nonfasting serum glucose of ≥200 mg/dL, taking medication for diabetes or high blood glucose, or self-reported diabetes diagnosis from a physician [to convert glucose to millimoles per liter, multiply by 0.0555]).

### Statistical Analysis

Descriptive statistics were calculated to describe the study population characteristics. Missingness of the neighborhood SES (approximately 25%) was primarily evident in young adulthood due to lower proportion of reported addresses successfully geocoded during this life epoch and incomplete census tract coverage in the 1960s.^25^ For covariates, the missingness was on average 13.3%. Therefore, we applied multiple imputation by chained equations with the life course variables and all covariates using a minimum of 10 imputed datasets created to impute the missing data.^26,27^

We estimated premature death rates stratified by life epoch neighborhood SES score using Poisson regression adjusting for age, sex, race, and study center with log person-years as an offset. Kaplan-Meier analysis was used to plot cumulative mortality by tertiles of adulthood cumulative neighborhood SES score. Cox proportional hazard regression was used to examine the association between life course neighborhood SES and premature mortality. In model 1, analyses were adjusted for age, sex, and study center. We further examined how sociodemographic factors affect the association between neighborhood SES and premature mortality; therefore, in model 2 we adjusted for model 1 and included marital status, race, and income. All other covariates were assessed but not included in the model as they did not materially alter the association between neighborhood SES and premature mortality. *P* for trend was calculated by modeling tertiles of neighborhood SES as a continuous variable. We further evaluated the interactions between neighborhood SES and sex, race, and individual SEP using the likelihood ratio test comparing models with and without the cross-product term.

Sensitivity analysis was performed to evaluate the association between neighborhood SES at each life epoch and premature death defined at age 70 years overall and stratified by race and sex. Analysis was performed using R version 4.0.2 (R Project for Statistical Computing) and SAS version 9.4 (SAS Institute). Two-sided *P* < .05 was considered statistically significant

## Results

A total of 12 610 participants were included; the mean (SD) age at study baseline was 62.6 (5.6) years; and 3181 (25.2%) were Black and 9429 (74.8%) were White. Men (5388 [42.7%]) were more likely than women (7222 [57.3%]) to have prevalent diabetes and to be a current tobacco smoker (Table 1). Women had lower neighborhood SES and individual SEP scores at each life epoch compared with men. When stratified by middle adulthood neighborhood SES, those in the lowest tertile were more likely to be Black, to have less than a high school education, and to have an annual income of less than $25 000 (eTable 2 in Supplement 1).

### Life Epoch Neighborhood SES

Over the course of a mean (SD) follow-up of 18.8 (5.7) years, there were 1605 premature deaths. The premature mortality rate was highest for the lowest neighborhood SES tertile in middle and young adulthood (eFigure 2 in Supplement 1). When comparing participants in the highest tertile of neighborhood SES at each life epoch, those in the lowest tertile had a higher risk of premature mortality in middle (HR, 1.59; 95% CI, 1.34–1.90) and young (HR, 1.32; 95% CI, 1.13–1.54) adulthood (Table 2). Results attenuated following adjustment for additional sociodemographic factors but remained statistically significant in middle adulthood. No significant association was observed between childhood neighborhood SES and premature mortality. In sex-stratified analysis, compared with the highest tertile in middle adulthood, the lowest tertile had a greater premature mortality risk among men (HR, 1.49; 95% CI, 1.17–1.89) and women (HR, 1.72; 95% CI, 1.34–2.21). Following adjustment for additional sociodemographic factors, this association remained significant only among women (HR, 1.39; 95% CI, 1.07–1.81; *P* for interaction < .001). In analysis stratified by race, compared with the highest neighborhood SES tertile in middle adulthood, the lowest tertile was associated with increased premature mortality risk among Black (HR, 1.67; 95% CI, 1.18–2.38) and White (HR, 1.48; 95% CI, 1.18–1.86) participants. After further adjustment, the association between premature mortality and the lowest tertile of neighborhood SES in middle adulthood was largely similar between racial groups (Black: HR, 1.24; 95% CI, 0.87–1.79; White: HR, 1.26; 95% CI, 1.00–1.59; *P* for interaction = .42). However, association with childhood neighborhood SES was only significant among White participants (Table 3).

### Adulthood Cumulative Neighborhood SES

The cumulative risk curves show that those in the lowest tertile of adulthood cumulative neighborhood SES score had higher likelihood of premature mortality throughout follow-up (eFigure 3 in Supplement 1). Compared with participants in the highest tertile of cumulative neighborhood SES, those in the lowest tertile had an increased premature mortality risk (HR, 1.49; 95% CI, 1.26–1.76) (eTable 3 in Supplement 1). Adjusting for additional sociodemographic factors, the association attenuated modestly (HR, 1.20; 95% CI, 1.01–1.43). Comparable estimates were observed among men and women; however, after adjustment for additional sociodemographic factors, the association was stronger among women (HR, 1.29; 95% CI, 1.01–1.67; *P* for interaction < .001). When examined by race, compared with the highest cumulative neighborhood SES tertile, lower tertiles were associated with increased premature mortality risk among Black (HR, 1.55; 95% CI, 1.14–2.10) and White (HR, 1.28; 95% CI, 1.02–1.60) participants. Adjusting for additional sociodemographic factors, no significant associations were observed (eTable 4 in Supplement 1).

### Individual SEP and Neighborhood SES Interaction

When the association between tertiles of neighborhood SES and premature mortality was examined within tertiles of individual SEP in middle adulthood, a significant result was only observed among individuals in the lowest tertile of individual SEP, with the lowest neighborhood SES tertile having an increased premature mortality risk relative to those in the highest neighborhood SES tertile (Table 4). Adjusting for additional sociodemographic factors, the association was larger among those in the lowest tertile of neighborhood SES and of individual SEP group in middle adulthood (HR, 1.53; 95% CI, 1.19–1.96; *P* for interaction = .02).

We further assessed patterns of change in neighborhood SES scores across adulthood life epochs in relation to premature mortality (Table 5). When compared with the stable-high neighborhood SES group, premature mortality risk was consistently higher for all other groups. Adjusting for additional sociodemographic factors, the association attenuated but remained, with a slightly greater risk for the stable-low neighborhood SES group (HR, 1.25; 95% CI, 1.05–1.49). Among both men and women, compared with stable-high neighborhood SES group, the stable-low group had the largest premature mortality relative risk. Adjusting for additional sociodemographic factors, increased premature mortality risk was only significant among men in the low-to-high group (HR, 1.32; 95% CI, 1.04–1.68; *P* for interaction < .001). In race-stratified analysis, compared with stable-high neighborhood SES, all other groups had significantly elevated risk of premature mortality among White participants but not Black participants (eTable 5 in Supplement 1). For example, when compared with those in the stable-high group, White participants in all other groups had an increased premature mortality risk, and the association remained after adjusting for additional sociodemographic factors.

### Sensitivity Analyses

In sensitivity analyses using age 70 years as the cutoff for premature mortality, the association between neighborhood SES in middle adulthood and risk of premature mortality attenuated in the overall population and by sex (eTable 6 in Supplement 1). Similar results were observed in race-stratified analysis (eTable 7 in Supplement 1).

## Discussion

In this large cohort of community-dwelling adults, we observed that low neighborhood SES across adulthood was associated with risk of premature mortality. This risk persisted after adjustment for sociodemographic factors. Our findings showed that the associated risk was greater among women and largely similar between racial groups. Additionally, the association between life course neighborhood SES and premature mortality was modified by individual SEP, with the strongest associations observed among individuals with the lowest level of individual SEP. We further found an association between changes in neighborhood SES between adulthood life epochs and risk of premature mortality.

Despite the importance of neighborhood SES to health over the life course, evaluations of the association between early life (childhood and young adulthood) neighborhood SES and premature mortality are sparse. Most prior studies evaluating neighborhood SES and mortality examined the association at 1 time point in middle to older adulthood, showing an increased mortality risk among those living in socioeconomically disadvantaged neighborhoods, aligning with present study findings.^11,12,28–30^ Our focus on life course neighborhood SES adds an important contextual understanding of how low early life neighborhood SES may affect risk of premature mortality. For instance, our finding that low neighborhood SES in young adulthood was associated with the risk of premature mortality suggests that early adulthood neighborhood socioeconomic conditions may have a lasting impact on health.^23,31–34^ Our findings align with the few extant studies that suggest an association of early life low neighborhood SES with cardiovascular disease risk and events in later life.^22,35,36^

We observed that the associations between young adulthood and middle adulthood neighborhood SES and premature mortality were stronger among women than men, consistent with evidence that suggests that women benefit more than men from residing in less deprived neighborhoods.^12,37^ Compared with men, women who reside in socioeconomically deprived neighborhoods experience greater neighborhood-related psychosocial stress and barriers to opportunities for socioeconomic mobility, both of which are associated with mortality.^1,22,38,39^ The association between neighborhood SES and premature mortality was largely similar between racial groups. However, the association between low childhood neighborhood SES and risk of premature mortality was only observed among White participants. Prior studies have suggested that because of racial residential segregation, Black individuals residing in socioeconomically disadvantaged neighborhoods are more likely to utilize their own social capital to provide resources to their children to buffer against adverse neighborhood conditions, and therefore the measure of neighborhood SES may not reflect the actual neighborhood exposure.^1,40–43^

Research on the interaction between individual SEP and neighborhood SES on health remains limited. As in our study, results from a prior population-based cohort study suggest a stronger gradient in the association between individual socioeconomic disadvantage and health-related lifestyle risk factors for individuals living in the most socioeconomic disadvantaged neighborhoods.^44^ A potential underpinning mechanism for these findings is that high individual SEP may reduce the reliance on the contextual socioeconomic conditions for accessing health resources.^1,3^

We extended previous research on the association of neighborhood SES with mortality by demonstrating that persistent exposure to low neighborhood SES from young to middle adulthood was associated with greater premature mortality risk compared with those who resided in stable-high neighborhood SES, although an elevated risk was observed for low neighborhood SES in either young or middle adulthood. Interestingly, low to high neighborhood SES had a slightly stronger association with premature mortality than high to low, suggesting low neighborhood SES in young adulthood has a greater influence on premature mortality risk. Our results are similar to previous studies that suggest that persistently low neighborhood SES across adult life stages is associated with increased risk of cardiovascular disease and mortality.^22,37^

### Limitations

Several limitations must be noted. First, Black participants were largely clustered within 1 study site; therefore, comparison between racial groups may be confounded by study center. Second, the LCSES study enrolled participants between 2001 and 2002 and not from ARIC study baseline; therefore, excluding participants who died prior enrollment may have introduced survival bias potentially influencing our estimates. Third, incomplete coverage of census tracts for earlier decades contributed to greater missing data for young adulthood neighborhood SES.^19^ However, we applied multiple imputation methods to address missing data, as this approach is recommended for reducing bias.^27,45^ Fourth, participants’ childhood address information could only be geocoded to the county level; therefore, comparisons between childhood and adulthood neighborhood SES (which was obtained at the census tract level) were not possible. Fifth, neighborhood address and individual socioeconomic information in childhood and young and middle adulthood are susceptible to recall bias.

## Conclusions

In this cohort study, we observed an association of sustained low adulthood neighborhood SES with the risk of premature mortality. Importantly, results of this study suggest that the risk of premature mortality was greatest among those experiencing combined low individual SEP and low neighborhood SES. Future studies aimed at identifying place-based interventions that target neighborhood social determinants of health should be designed from a life course perspective that accounts for early-life socioeconomic inequality, as this is a critical route to alleviating premature death.

## Supplementary Material

Supplement Online Content 1. **eFigure 1.** Timeline of the Atherosclerosis Risk in Communities Study
**eFigure 2.** Adjusted Premature Death Rates (per 1000 Person-Years)
**eFigure 3.** Cumulative Incidence of Premature Mortality Stratified by Tertile of Adulthood Cumulative Neighborhood Socioeconomic Status Score
**eTable 1.** Neighborhood and Individual Life-Course Socioeconomic Factors and Scoring, Atherosclerosis Risk in Communities Study, 1996–2020
**eTable 2.** Characteristics of Atherosclerosis Risk in Communities Study Participants by Tertile Middle Adulthood Neighborhood Socioeconomic Status, 1996–2020
**eTable 3.** Associations Between Adulthood Cumulative Neighborhood Socioeconomic Status and Premature Mortality Overall and Stratified by Sex, Atherosclerosis Risk in Communities Study, 1996–2020
**eTable 4.** Associations Between Adulthood Cumulative Neighborhood Socioeconomic Status and Premature Mortality Stratified by Racial Group, Atherosclerosis Risk in Communities Study, 1996–2020
**eTable 5.** Associations Between Neighborhood Socioeconomic Status Patterns Across Adulthood Life Epochs and Premature Mortality Stratified by Racial Group, Atherosclerosis Risk in Communities Study, 1996–2020
**eTable 6.** Associations Between Life Epoch Neighborhood Socioeconomic Status and Premature Mortality at Age 70 Cut-Off Overall and Stratified by Sex, Atherosclerosis Risk in Communities Study, 1996–2020
**eTable 7.** Associations Between Life Epoch Neighborhood Socioeconomic Status and Premature Mortality at Age 70 Cut-Off Stratified by Racial Group, Atherosclerosis Risk in Communities Study, 1996–2020

Supplement Online Content 2. Data Sharing Statement

## Figures and Tables

**Table 1.** Study Baseline Characteristics of Atherosclerosis Risk in Communities Study Participants Overall and Stratified by Sex, 1996–2020

	Participants, No. (%)		
Characteristic^a^	Overall (N = 12 610)	Men (n = 5388)	Women (n = 7222)
Age, mean (SD), y	62.6 (5.6)	63.0 (5.7)	62.4 (5.6)
Racial group			
Black	3181 (25.2)	1115 (20.7)	2066 (28.6)
White	9429 (74.8)	4273 (79.3)	5156 (71.4)
Education, <HS	2648 (21.0)	1103 (20.5)	1545 (21.4)
Married	8091 (76.1)	3990 (87.4)	4101 (67.6)
Annual family income <$25 000	3181 (31.1)	997 (22.5)	2184 (37.7)
Current tobacco smoker	1517 (14.3)	698 (15.3)	819 (13.5)
Current alcohol drinker	5288 (49.7)	2669 (58.5)	2619 (43.2)
Body mass index, mean (SD)^b^	28.9 (5.6)	28.5 (4.4)	29.2 (6.3)
Hypertension^c^	5045 (47.2)	2076 (45.4)	2969 (48.7)
Diabetes^d^	1709 (16.1)	787 (17.3)	922 (15.2)
Neighborhood SES across life epochs, mean (SD), *z* score^e^			
Middle adulthood	0.3 (4.8)	0.7 (4.6)	0.01 (4.8)
Young adulthood	0.7 (4.9)	1.04 (4.9)	0.5 (5.0)
Childhood	0.004 (4.02)	0.06 (4.0)	−0.04 (4.05)
Individual SEP across life epochs, mean (SD), score^e^			
Middle adulthood	2.4 (1.5)	2.9 (1.5)	2.0 (1.3)
Young adulthood	2.2 (1.3)	2.5 (1.4)	2.0 (1.1)
Childhood	2.0 (1.4)	2.0 (1.4)	1.9 (1.4)

Abbreviations: HS, high school; SEP, socioeconomic position; SES, socioeconomic status.

a Calculated using data without imputation.

b Calculated as weight in kilograms divided by height in meters squared.

c Hypertension was defined as systolic blood pressure of 140 mm Hg or greater, diastolic blood pressure of 90 mm Hg or greater, or use of medication to treat hypertension.

d Diabetes was defined as fasting blood glucose concentration 126 mg/dL or greater, self-reported diagnosis of diabetes, or use of medication to treat diabetes (to convert glucose to millimoles per liter, multiply by 0.0555).

e Middle adulthood was defined as age 40 to 50 years; young adulthood, age 30 years; childhood, age 10 years.

**Table 2.** Associations Between Life Epoch Neighborhood SES and Premature Mortality Overall and Stratified by Sex, Atherosclerosis Risk in Communities Study, 1996–2020

	HR (95% CI)^b^					
	Overall (N = 12 610)		Men (n = 5388)		Women (n = 7222)	
Life epoch^a^	Model 1^c^	Model 2^d^	Model 1^e^	Model 2^f^	Model 1^e^	Model 2^f^
**Middle adulthood neighborhood SES** ^g^						
T1 (lowest)	1.59 (1.34–1.90)	1.28 (1.07–1.54)	1.49 (1.17–1.89)	1.19 (0.93–1.53)	1.72 (1.34–2.21)	1.39 (1.07–1.81)
T2	1.41 (1.21–1.63)	1.26 (1.08–1.46)	1.31 (1.07–1.60)	1.18 (0.96–1.44)	1.53 (1.23–1.91)	1.37 (1.09–1.71)
T3 (highest)	1 [Reference]	1 [Reference]	1 [Reference]	1 [Reference]	1 [Reference]	1 [Reference]
*P* for trend	<.001	.01	.001	.15	<.001	.02
**Young adulthood neighborhood SES** ^g^						
T1 (lowest)	1.32 (1.13–1.54)	1.16 (0.99–1.36)	1.22 (0.99–1.52)	1.08 (0.86–1.34)	1.41 (1.13–1.76)	1.25 (1.00–1.56)
T2	1.17 (1.01–1.35)	1.10 (0.95–1.27)	1.28 (1.06–1.55)	1.21 (1.00–1.47)	1.05 (0.85–1.30)	1.00 (0.80–1.23)
T3 (highest)	1 [Reference]	1 [Reference]	1 [Reference]	1 [Reference]	1 [Reference]	1 [Reference]
*P* for trend	<.001	.06	.05	.44	.002	.05
**Childhood neighborhood SES** ^g^						
T1 (lowest)	1.12 (0.96–1.32)	1.09 (0.93–1.28)	1.14 (0.91–1.42)	1.09 (0.87–1.36)	1.11 (0.88–1.39)	1.11 (0.88–1.39)
T2	1.05 (0.91–1.22)	1.06 (0.91–1.23)	1.05 (0.85–1.29)	1.04 (0.84–1.28)	1.06 (0.86–1.31)	1.09 (0.88–1.35)
T3 (highest)	1 [Reference]	1 [Reference]	1 [Reference]	1 [Reference]	1 [Reference]	1 [Reference]
*P* for trend	.16	.26	.26	.48	.39	.35

Abbreviations: HR, hazard ratio; T, tertile; SES, socioeconomic status.

a Middle adulthood was based on age 40 to 50 years; young adulthood, age 30 years; childhood, age 10 years.

b There were 1605 premature deaths overall, with 816 among men and 789 among women.

c Model 1 was adjusted for age (years; continuous), sex (male or female), and study center (Washington County, Maryland; Forsyth County, North Carolina; Jackson, Mississippi; or northwestern suburbs of Minneapolis, Minnesota).

d Model 2 was adjusted for Model 1 as well as marital status (married, widowed, divorced, separated, or never married), racial group (Black participants or White participants), and annual household income (<$25 000, $25 000–$49 999, $50 000–$74 999, or ≥$75 000).

e Model 1 was adjusted for age (years; continuous) and study center (Washington County, Maryland; Forsyth County, North Carolina; Jackson, Mississippi; or northwestern suburbs of Minneapolis, Minnesota).

f Model 2 was adjusted for Model 1 as well as marital status (married, widowed, divorced, separated, or never married), racial group (Black participants or White participants), and annual household income (<$25 000, $25 000–$49 999, $50 000–$74 999, or ≥$75 000).

g *P* for interaction, based on model 2, for all life epochs was <.001.

**Table 3.** Associations Between Life Epoch Neighborhood SES and Premature Mortality Stratified by Racial Group, Atherosclerosis Risk in Communities Study, 1996–2020

	HR (95% CI)^b^			
	Black participants (n = 3181)		White participants (n = 9429)	
Life epoch^a^	Model 1^c^	Model 2^d^	Model 1^c^	Model 2^d^
**Middle adulthood neighborhood SES** ^e^				
T1 (low)	1.67 (1.18–2.38)	1.24 (0.87–1.79)	1.48 (1.18–1.86)	1.26 (1.00–1.59)
T2	1.38 (0.94–2.04)	1.17 (0.79–1.73)	1.42 (1.21–1.67)	1.31 (1.11–1.54)
T3 (high)	1 [Reference]	1 [Reference]	1 [Reference]	1 [Reference]
*P* for trend	.002	.24	<.001	.01
**Young adulthood neighborhood SES** ^f^				
T1 (low)	1.47 (1.10–1.95)	1.31 (0.98–1.75)	1.20 (0.98–1.46)	1.08 (0.88–1.31)
T2	1.20 (0.87–1.65)	1.19 (0.86–1.64)	1.17 (1.00–1.38)	1.10 (0.93–1.29)
T3 (high)	Reference	Reference	Reference	Reference
*P* for trend	.01	.07	.05	.40
**Childhood neighborhood SES** ^g^				
T1 (low)	1.01 (0.79–1.29)	1.00 (0.78–1.28)	1.31 (1.05–1.62)	1.25 (1.01–1.56)
T2	1.11 (0.86–1.44)	1.13 (0.87–1.46)	1.05 (0.87–1.26)	1.05 (0.88–1.27)
T3 (high)	Reference	Reference	Reference	Reference
*P* for trend	.99	.89	.02	.05

Abbreviations: HR, hazard ratio; T, tertile; SES, socioeconomic status.

a Middle adulthood was defined as age 40 to 50 years; young adulthood, age 30 years; childhood, age 10 years.

b There were a total of 1605 premature deaths, with 584 among Black participants and 1021 among White participants.

c Model 1 was adjusted for age (years; continuous), sex (male or female), and study center (Washington County, Maryland; Forsyth County, North Carolina; Jackson, Mississippi; or northwestern suburbs of Minneapolis, Minnesota).

d Model 2 was adjusted for Model 1 as well as marital status (married, widowed, divorced, separated, or never married) and annual household income (<$25 000, $25 000–$49 999, $50 000–$74 999, or ≥$75 000).

e *P* for interaction, based on model 2, for middle adulthood neighborhood SES and race was .42.

f *P* for interaction, based on model 2, for young adulthood neighborhood SES and race was .18.

g *P* for interaction, based on model 2, for childhood neighborhood SES and race was .22.

**Table 4.** Associations Between Life Epoch Neighborhood SES and Premature Mortality by Life Epoch Individual SEP, Atherosclerosis Risk in Communities Study, 1996–2020

	HR (95% CI)					
	T1 (lowest) individual SEP		T2 individual SEP		T3 (highest) individual SEP	
Neighborhood SES	Model 1^a^	Model 2^b^	Model 1^a^	Model 2^b^	Model 1^a^	Model 2^b^
**Middle adulthood** ^c^						
Total No.	6436	6436	4167	4167	2007	2007
T1 (lowest)	1.74 (1.36–2.21)	1.53 (1.19–1.96)	1.01 (0.70–1.45)	0.92 (0.63–1.33)	1.01 (0.58–1.76)	0.76 (0.43–1.36)
T2	1.37 (1.09–1.72)	1.30 (1.04–1.64)	1.28 (1.01–1.63)	1.23 (0.96–1.57)	1.47 (1.03–2.11)	1.32 (0.91–1.90)
T3 (highest)	1 [Reference]	1 [Reference]	1 [Reference]	1 [Reference]	1 [Reference]	1 [Reference]
*P* for trend	<.001	.001	.43	.82	.41	.85
**Young adulthood** ^d^						
Total No.	7938	7938	2706	2706	1966	1966
T1 (lowest)	1.30 (1.07–1.57)	1.19 (0.98–1.44)	0.97 (0.65–1.45)	0.94 (0.63–1.42)	0.94 (0.59–1.48)	0.88 (0.56–1.40)
T2	1.12 (0.93–1.35)	1.08 (0.89–1.30)	1.12 (0.82–1.54)	1.12 (0.82–1.54)	1.15 (0.82–1.61)	1.10 (0.79–1.54)
T3 (highest)	1 [Reference]	1 [Reference]	1 [Reference]	1 [Reference]	1 [Reference]	1 [Reference]
*P* for trend	.01	.07	.96	.95	.99	.78
**Childhood** ^e^						
Total No.	5746	5746	3053)	3053	3811	3811
T1 (lowest)	1.17 (0.95–1.45)	1.16 (0.93–1.44)	1.06 (0.76–1.47)	1.05 (0.75–1.45)	0.96 (0.67–1.37)	0.94 (0.66–1.35)
T2	1.15 (0.93–1.43)	1.15 (0.93–1.43)	0.90 (0.67–1.22)	0.95 (0.70–1.28)	1.01 (0.76–1.34)	1.00 (0.75–1.34)
T3 (highest)	1 [Reference]	1 [Reference]	1 [Reference]	1 [Reference]	1 [Reference]	1 [Reference]
*P* for trend	.14	.19	.81	.83	.84	.78

Abbreviations: HR, hazard ratio; T, tertile; SEP, socioeconomic position; SES, socioeconomic status.

a Model 1 was adjusted for age (years; continuous), sex (male or female), and study center (Washington County, Maryland; Forsyth County, North Carolina; Jackson, Mississippi; or northwestern suburbs of Minneapolis, Minnesota).

b Model 2 was adjusted for Model 1 as well as marital status (married, widowed, divorced, separated, or never married), racial group (Black participants or White participants), and annual household income (<$25 000, $25 000–$49 999, $50 000–$74 999, or ≥$75 000).

c Middle adulthood was define as age 40 to 50 years. By individual SEP, there were 951 premature deaths in T1; 464 in T2; and 190 in T3. *P* for interaction, based on model 2, was .02.

d Young adulthood was defined as age 30 years. By individual SEP, there were 1111 premature deaths in T1, 268 in T2, and 226 in T3. *P* for interaction, based on model 2, was .02.

e Childhood was defined as age 10 years. By individual SEP, there were 839 premature deaths in T1, 383 in T2, and 383 in T3. *P* for interaction, based on model 2, was .06.

**Table 5.** Associations Between Neighborhood Socioeconomic Status Patterns Across Adulthood Life Epoch and Premature Mortality in the Overall Population and Stratified by Sex, Atherosclerosis Risk in Communities Study, 1996–2020

	Overall (N = 12 610)			Men (n = 5388)			Women (n = 7222)		
Young-to-middle adulthood neighborhood SES^a^		HR (95% CI)^b^			HR (95% CI)^b^			HR (95% CI)^b^	
	No.	Model 1^c^	Model 2^d^	No.	Model 1^e^	Model 2^f^	No.	Model 1^e^	Model 2^f^
Low-low neighborhood SES	4584	1.52 (1.28–1.79)	1.25 (1.05–1.49)	1803	1.49 (1.18–1.87)	1.25 (0.99–1.59)	2781	1.54 (1.20–1.96)	1.25 (0.97–1.61)
Low-high neighborhood SES	1721	1.31 (1.09–1.57)	1.22 (1.02–1.46)	756	1.40 (1.10–1.78)	1.32 (1.04–1.68)	965	1.21 (0.92–1.59)	1.12 (0.85–1.47)
High-low neighborhood SES	1719	1.34 (1.10–1.63)	1.16 (0.95–1.42)	747	1.33 (1.02–1.75)	1.20 (0.91–1.58)	972	1.34 (1.01–1.80)	1.12 (0.83–1.50)
High-high neighborhood SES	4586	1 [Reference]	1 [Reference]	2082	1 [Reference]	1 [Reference]	2504	1 [Reference]	1 [Reference]

Abbreviations: HR, hazard ratio; SES, socioeconomic status.

a Middle adulthood was defined as age 40 to 50 years; young adulthood, age 30 years. Low and high neighborhood SES was defined based on the median neighborhood SES at each life stage. *P* for interaction, based on Model 2, was <.001.

b There were 1605 total events, with 816 among men and 789 among women.

c Model 1 was adjusted for age (years; continuous), sex (male or female), and study center (Washington County, Maryland; Forsyth County, North Carolina; Jackson, Mississippi; or northwestern suburbs of Minneapolis, Minnesota).

d Model 2 was adjusted for Model 1 as well as marital status (married, widowed, divorced, separated, or never married) and annual household income (<$25 000, $25 000–$49 999, $50 000–$74 999, or ≥$75 000).

e Model 1 was adjusted for age (years; continuous) and study center (Washington County, Maryland; Forsyth County, North Carolina; Jackson, Mississippi; or northwestern suburbs of Minneapolis, Minnesota).

f Model 2 was adjusted for Model 1 as well as marital status (married, widowed, divorced, separated, or never married), racial group (Black participant or White participant), and annual household income (<$25 000, $25 000–$49 999, $50 000–$74 999, or ≥$75 000).

## Data Availability

See Supplement 2.

## Key Points

### Question

Is lower life course neighborhood socioeconomic status (SES) associated with premature mortality?

### Findings

In this cohort study of 12 610 men and women, low life course neighborhood SES, experienced particularly during the period from young to middle adulthood, was associated with increased risk of premature mortality. Estimates were larger among women.

### Meaning

In this study, low neighborhood SES across the life course was associated with premature mortality.
