# Supplementary material for: Neighborhood Socioeconomic Disadvantage Across the Life Course and Premature Mortality
Source: JAMA Netw Open. Author manuscript; Available in PMC 2024 Aug 19. (PMC11307131; doi:10.1001/jamanetworkopen.2024.26243)
Supplement: Supplement Online Content 1. — eFigure 1. Timeline of the Atherosclerosis Risk in Communities Study eFigure 2. Adjusted Premature Death Rates (per 1000 Person-Years) eFigure 3. Cumulative Incidence of Premature Mortality Stratified by Tertile of Adulthood Cumulative Neighborhood Socioeconomic Status Score eTable 1. Neighborhood and Individual Life-Course Socioeconomic Factors and Scoring, Atherosclerosis Risk in Communities Study, 1996–2020 eTable 2. Characteristics of Atherosclerosis Risk in Communities Study Participants by Tertile Middle Adulthood Neighborhood Socioeconomic Status, 1996–2020 eTable 3. Associations Between Adulthood Cumulative Neighborhood Socioeconomic Status and Premature Mortality Overall and Stratified by Sex, Atherosclerosis Risk in Communities Study, 1996–2020 eTable 4. Associations Between Adulthood Cumulative Neighborhood Socioeconomic Status and Premature Mortality Stratified by Racial Group, Atherosclerosis Risk in Communities Study, 1996–2020 eTable 5. Associations Between Neighborhood Socioeconomic Status Patterns Across Adulthood Life Epochs and Premature Mortality Stratified by Racial Group, Atherosclerosis Risk in Communities Study, 1996–2020 eTable 6. Associations Between Life Epoch Neighborhood Socioeconomic Status and Premature Mortality at Age 70 Cut-Off Overall and Stratified by Sex, Atherosclerosis Risk in Communities Study, 1996–2020 eTable 7. Associations Between Life Epoch Neighborhood Socioeconomic Status and Premature Mortality at Age 70 Cut-Off Stratified by Racial Group, Atherosclerosis Risk in Communities Study, 1996–2020 [file NIHMS2015532-supplement-Supplement_Online_Content_1_.pdf]

Supplemental Online Content

Lawrence WR, Kucharska-Newton AM, Magnani JW, et al. Neighborhood socioeconomic disadvantage across the life course and premature mortality. *JAMA Netw Open.* 2024;7(8):e2426243. doi:10.1001/jamanetworkopen.2024.26243

eFigure 1. Timeline of the Atherosclerosis Risk in Communities Study

eFigure 2. Adjusted Premature Death Rates (per 1000 Person-Years)

eFigure 3. Cumulative Incidence of Premature Mortality Stratified by Tertile of Adulthood Cumulative Neighborhood Socioeconomic Status Score

eTable 1. Neighborhood and Individual Life-Course Socioeconomic Factors and Scoring, Atherosclerosis Risk in Communities Study, 1996-2020

eTable 2. Characteristics of Atherosclerosis Risk in Communities Study Participants by Tertile Middle Adulthood Neighborhood Socioeconomic Status, 1996-2020

eTable 3. Associations Between Adulthood Cumulative Neighborhood Socioeconomic Status and Premature Mortality Overall and Stratified by Sex, Atherosclerosis Risk in Communities Study, 1996-2020

eTable 4. Associations Between Adulthood Cumulative Neighborhood Socioeconomic Status and Premature Mortality Stratified by Racial Group, Atherosclerosis Risk in Communities Study, 1996-2020

eTable 5. Associations Between Neighborhood Socioeconomic Status Patterns Across Adulthood Life Epochs and Premature Mortality Stratified by Racial Group, Atherosclerosis Risk in Communities Study, 1996-2020

eTable 6. Associations Between Life Epoch Neighborhood Socioeconomic Status and Premature Mortality at Age 70 Cut-Off Overall and Stratified by Sex, Atherosclerosis Risk in Communities Study, 1996-2020

eTable 7. Associations Between Life Epoch Neighborhood Socioeconomic Status and Premature Mortality at Age 70 Cut-Off Stratified by Racial Group, Atherosclerosis Risk in Communities Study, 1996-2020

This supplemental material has been provided by the authors to give readers additional information about their work.

© 2024 Lawrence WR et al. *JAMA Network Open.*

eFigure 1. Timeline of the Atherosclerosis Risk in Communities Study

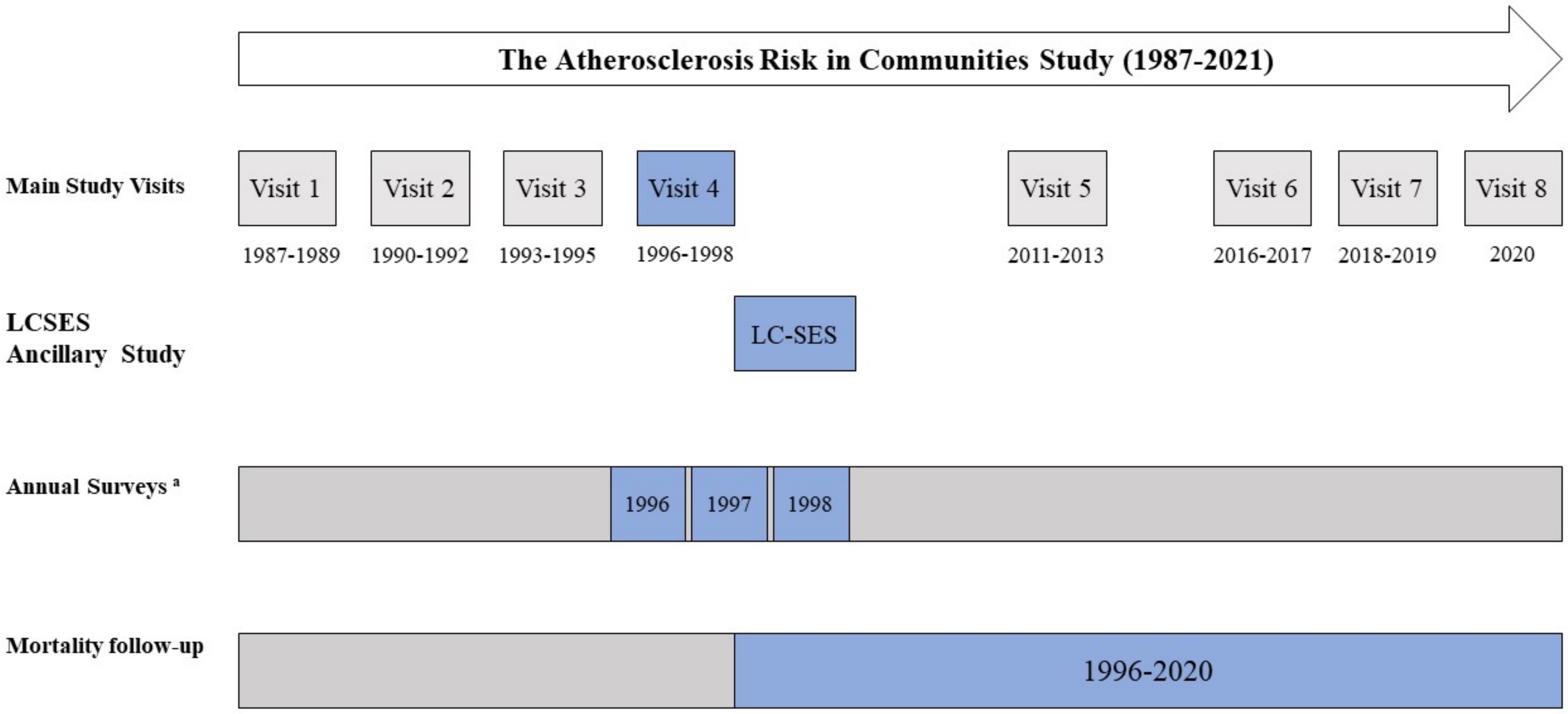

Abbreviations: LCSES, Life-Course Socioeconomic Status
[a] Since 2012, surveys have been conducted semi-annually.

Note: Blue boxes indicate visits, surveys, and the follow-up period from which from which variables were collected and then used in analyses.

© 2024 Lawrence WR et al. *JAMA Network Open.*

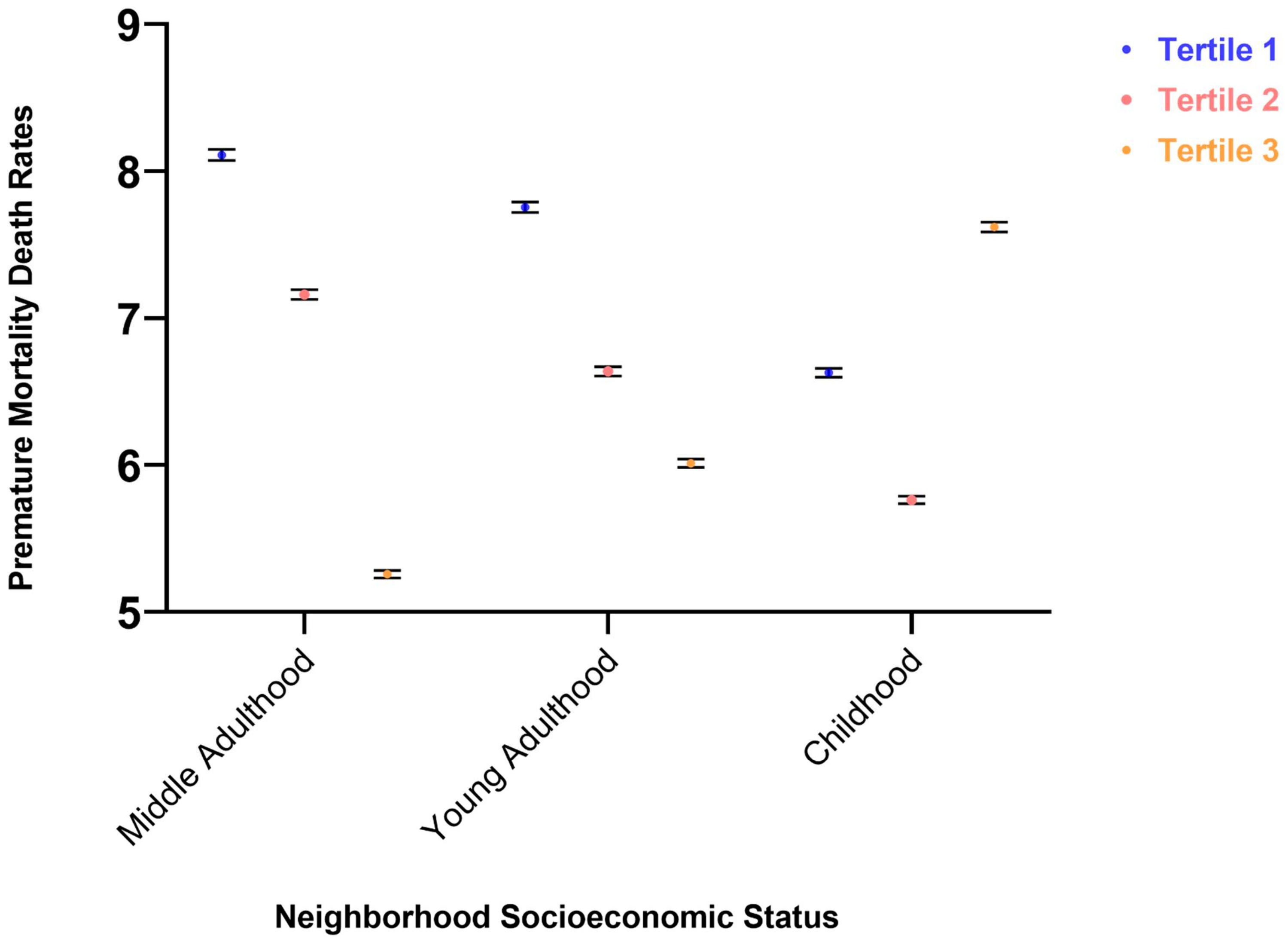

eFigure 2. Adjusted Premature Death Rates (per 1000 Person-Years)
Premature Death rates adjusted for age (years; continuous), sex (male or female), racial group (Black or White), and study center (Washington County, Maryland; Forsyth County, North Carolina; Jackson, Mississippi; or northwestern suburbs of Minneapolis, Minnesota).
Note: Middle adulthood: age 40–50 years; young adulthood: age 30 years; childhood: age 10 years.

© 2024 Lawrence WR et al. *JAMA Network Open.*

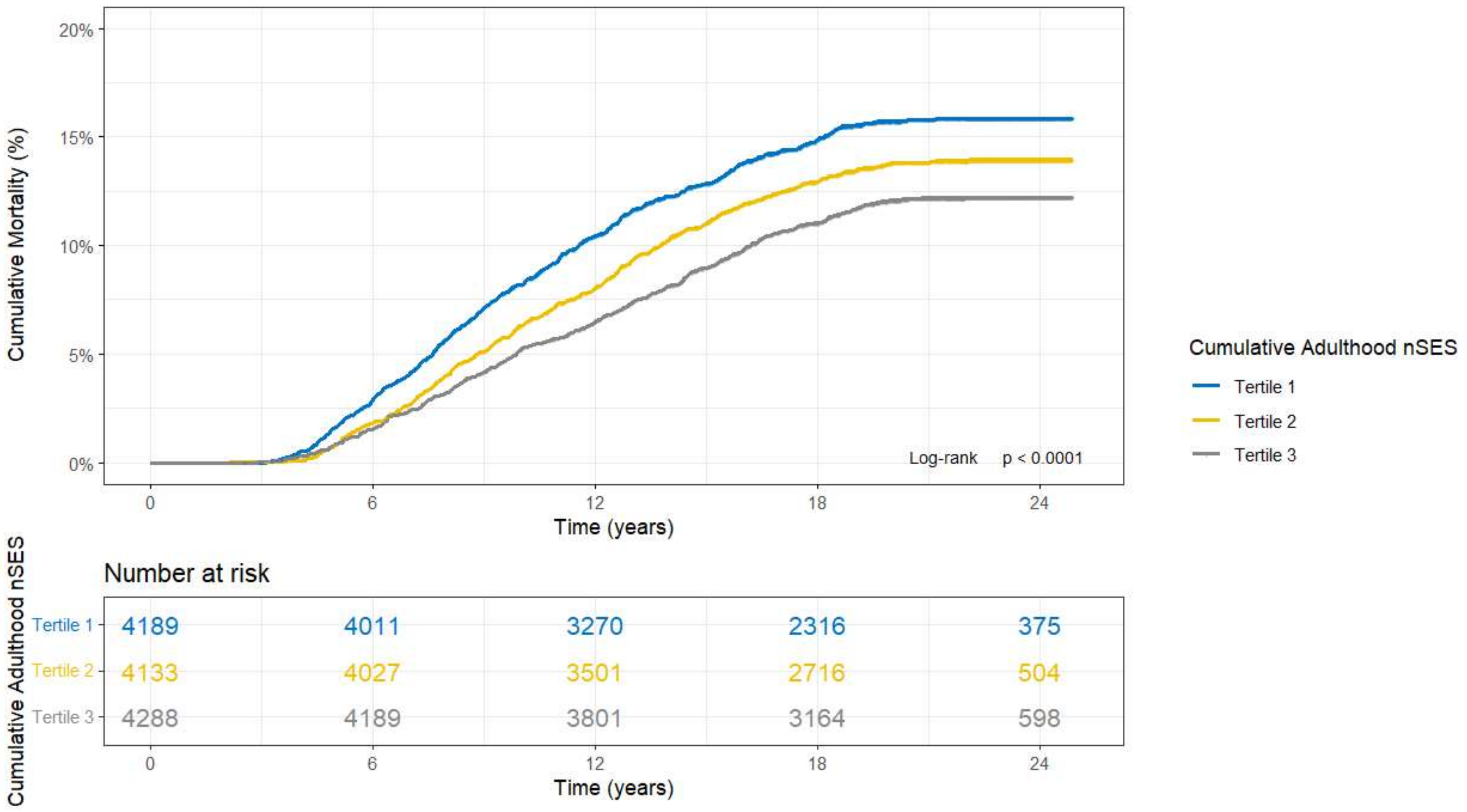

eFigure 3. Cumulative Incidence of Premature Mortality Stratified by Tertile of Adulthood Cumulative Neighborhood Socioeconomic Status Score

Abbreviation: nSES, neighborhood socioeconomic status.
Note: Adulthood Cumulative nSES is the sum of neighborhood socioeconomic status z-scores in young adulthood and middle adulthood.
Tertile 1=low neighborhood socioeconomic status score and Tertile 3=high neighborhood socioeconomic status score

© 2024 Lawrence WR et al. *JAMA Network Open.*

eTable 1. Neighborhood and Individual Life-Course Socioeconomic Factors and Scoring, Atherosclerosis Risk in Communities Study, 1996-2020

Neighborhood Life-Course Socioeconomic Status		Individual Life-Course Socioeconomic Status	
Life Stages	z-Score [a]	Variable	Score
Childhood (Age 10 Years)			
Adult education	Proportion with high-school or college degree	Parental education	≤8th grade = 0<br>9 to 11 grades = 1<br>≥12 grade = 2
Adult occupational role	Proportion with managerial roles	Parental occupation	Manual = 0<br>Nonmanual = 1
Dwellings occupied by owner	Proportion of homes occupied by owner	Parental occupational role	Managerial = 1<br>Nonmanagerial = 0
Log median home value	Median value of homes	Parental home ownership	Rent or other = 0<br>Own home = 1
Young Adulthood (Age 30 Years)			
Adult education	Proportion with high-school or college degree	Education	Less than high school = 0<br>High school = 1<br>Beyond high school = 2
Adult occupational role	Proportion with managerial roles	Occupation	Manual = 0<br>Nonmanual = 1
Log median income	Median family income	Occupational role	Nonmanagerial = 0<br>Managerial = 1
Dwellings occupied by owner	Proportion of homes occupied by owner	Home ownership	Rent or other = 0<br>Own home = 1
Log median home value	Median value of homes		
Middle Adulthood (Ages 40–50 Years)			
Adult education	Proportion with high-school or college degree	Income, $ [b]	<25,000 = 0<br>25,000–34,999 = 1<br>>35,000 = 2
Adult occupational role	Proportion with managerial roles	Occupation	Manual = 0<br>Non-manual = 1
Log median income	Median family income	Occupational role	Non-managerial = 0<br>Managerial = 1
Dwellings occupied by owner	Proportion of homes occupied by owner	Home ownership	Rent or other = 0<br>Own home = 1
Log median home value	Median value of homes		

[a] Values for z scores derived from census-tract data representing the location a participant reported living during each life epoch.
[b] Middle adulthood individual income based on ages 45-64

© 2024 Lawrence WR et al. *JAMA Network Open.*

eTable 2. Characteristics of Atherosclerosis Risk in Communities Study Participants by Tertile Middle Adulthood Neighborhood Socioeconomic Status, 1996-2020

	Tertile of Neighborhood SES in Middle Adulthood [b]		
Characteristics [a]	Tertile 1<br>-15.50 to -2.24	Tertile 2<br>-2.24 to 1.53	Tertile 3<br>1.53 to 14.32
Age, years, mean (SD)	64.4 (5.7)	62.5 (5.4)	60.9 (5.2)
Racial Group			
Black, n (%)	2,221 (69.8)	648 (20.4)	312 (9.8)
White, n (%)	1,940 (20.6)	3,513 (37.2)	3,976 (42.2)
Sex, female, n (%)	2,540 (35.1)	2,308 (32.0)	2,374 (32.9)
Education, less that high school, n (%)	1,623 (61.2)	761 (28.7)	269 (10.1)
Married, n (%)	2,547 (27.1)	3,281 (35.0)	3,562 (37.9)
Annual family income under $25,000, n (%)	2,425 (57.3)	1,241 (29.4)	564 (13.3)
Current tobacco smoker, n (%)	687 (35.6)	651 (33.8)	591 (30.6)
Current alcohol drinker, n (%)	1,207 (19.9)	2,043 (33.7)	2,813 (46.4)
Body mass index, mean (SD) [b]	29.9 (6.2)	28.9 (5.5)	28.1 (5.2)
Hypertension, n (%) [c]	2,518 (41.3)	1,956 (32.0)	1,632 (26.7)
Diabetes, n (%) [d]	1,011 (46.6)	652 (30.1)	505 (23.3)
Middle adulthood individual SEP: Score, mean (SD) [b]	1.6 (1.3)	2.4 (1.3)	2.9 (1.2)

Abbreviations: SD, standard deviation; SES, socioeconomic status; SEP, socioeconomic position; HS, high school

[a] Calculated using imputed dataset.

[b] Calculated as weight (kg)/height (m)2.

[c] Hypertension was defined as systolic blood pressure ≥140 mm Hg, diastolic blood pressure ≥90 mm Hg, or use of medication to treat hypertension.

[d] Diabetes was defined as fasting blood glucose concentration ≥126 mg/dL, self-reported diagnosis of diabetes, or use of medication to treat diabetes.

[e] Middle adulthood: age 40–50 years; young adulthood: age 30 years; childhood: age 10 years.

© 2024 Lawrence WR et al. *JAMA Network Open.*

eTable 3. Associations Between Adulthood Cumulative Neighborhood Socioeconomic Status and Premature Mortality Overall and Stratified by Sex, Atherosclerosis Risk in Communities Study, 1996-2020

	Overall (12,610)		Men (n=5,388)		Women (n=7,222)	
	Model 1 [a] HR (95% CI)	Model 2 [b] HR (95% CI)	Model 1 [c] HR (95% CI)	Model 2 [d] HR (95% CI)	Model 1 [c] HR (95% CI)	Model 2 [d] HR (95% CI)
Adulthood cumulative neighborhood SES [e]						
T1 (low)	1.49 (1.26-1.76)	1.20 (1.01-1.43)	1.39 (1.10-1.76)	1.12 (0.87-1.42)	1.59 (1.25-2.03)	1.29 (1.01-1.67)
T2	1.19 (1.03-1.39)	1.06 (0.91-1.24)	1.18 (0.97-1.45)	1.07 (0.87-1.31)	1.21 (0.97-1.51)	1.06 (0.85-1.33)
T3 (high)	Reference	Reference	Reference	Reference	Reference	Reference
P_{trend}	<0.001	0.04	0.01	0.38	<0.001	0.04
$P_{interaction}$	<0.001					

Abbreviations: CI, confidence interval; HR, hazard ratio; T, tertile; SES, socioeconomic status.
[a] Model 1: adjusted for age (years; continuous) sex (male or female), and study center (Washington County, Maryland; Forsyth County, North Carolina; Jackson, Mississippi; or northwestern suburbs of Minneapolis, Minnesota).
[b] Model 2: adjusted for Model 1+ marital status (married, widowed, divorced, separated, or never married), racial group (Black participants or White participants), and annual household income (<$25,000, $25,000–$49,999, $50,000–$74,999, or ≥$75,000).
[c] Model 1: adjusted for age (years; continuous), and study center (Washington County, Maryland; Forsyth County, North Carolina; Jackson, Mississippi; or northwestern suburbs of Minneapolis, Minnesota).
[d] Model 2: adjusted for Model 1+ marital status (married, widowed, divorced, separated, or never married), racial group (Black or White), and annual household income (<$25,000, $25,000–$49,999, $50,000–$74,999, or ≥$75,000).
[e] Sum of neighborhood SES z-scores in young adulthood and middle adulthood.
Note: $P_{interaction}$ based on Model 2.
Number of events:
Overall n=1,605; Men n=816; Women: n=789.

© 2024 Lawrence WR et al. *JAMA Network Open.*

eTable 4. Associations Between Adulthood Cumulative Neighborhood Socioeconomic Status and Premature Mortality Stratified by Racial Group, Atherosclerosis Risk in Communities Study, 1996-2020

	Black Participants (n=3,181)		White Participants (n=9,429)	
	Model 1 [a] HR (95% CI)	Model 2 [b] HR (95% CI)	Model 1 [a] HR (95% CI)	Model 2 [b] HR (95% CI)
Adulthood cumulative neighborhood SES [c]				
T1 (low)	1.55 (1.14-2.10)	1.23 (0.90-1.69)	1.28 (1.02-1.60)	1.07 (0.85-1.35)
T2	0.95 (0.67-1.36)	0.86 (0.60-1.22)	1.25 (1.06-1.48)	1.13 (0.96-1.34)
T3 (high)	Reference	Reference	Reference	Reference
P_{trend}	<0.001	0.04	0.01	0.42
$P_{interaction}$	0.01			

Abbreviations: CI, confidence interval; HR, hazard ratio; T, tertile; SES, socioeconomic status.
[a] Model 1: adjusted for age (years; continuous) sex (male or female), and study center (Washington County, Maryland; Forsyth County, North Carolina; Jackson, Mississippi; or northwestern suburbs of Minneapolis, Minnesota).
[b] Model 2: adjusted for Model 1+ marital status (married, widowed, divorced, separated, or never married), and annual household income (<$25,000, $25,000–$49,999, $50,000–$74,999, or ≥$75,000).
[c] Sum of neighborhood SES z-scores in young adulthood and middle adulthood.
Note: $P_{interaction}$ based on Model 2.
Number of events:
Black participants n=584; White participants: n=1,021.

© 2024 Lawrence WR et al. *JAMA Network Open.*

eTable 5. Associations Between Neighborhood Socioeconomic Status Patterns Across Adulthood Life Epochs and Premature Mortality Stratified by Racial Group, Atherosclerosis Risk in Communities Study, 1996-2020

	Black Participants (n=3,181)			White Participants (n=9,429)		
		Model 1 [a]	Model 2 [b]		Model 1 [a]	Model 2 [b]
	n	HR (95% CI)	HR (95% CI)	*n*	HR (95% CI)	HR (95% CI)
Young-to-Middle adulthood neighborhood SES [c,d]						
Low-Low neighborhood SES	2,177	1.47 (1.00-2.17)	1.14 (0.77-1.68)	2,407	1.42 (1.16-1.74)	1.24 (1.01-1.52)
Low-High neighborhood SES	272	1.10 (0.67-1.81)	1.03 (0.63-1.69)	1,449	1.35 (1.11-1.65)	1.27 (1.04-1.54)
High-Low neighborhood SES	500	1.10 (0.70-1.71)	0.87 (0.56-1.37)	1,219	1.50 (1.19-1.90)	1.37 (1.09-1.73)
High-High neighborhood SES	232	Reference	Reference	4,354	Reference	Reference
$P_{interaction}$				0.16		

Abbreviations: CI, confidence interval; HR, hazard ratio; T, tertile; SES, socioeconomic status.

[a] Model 1: adjusted for age (years; continuous) sex (male or female), and study center (Washington County, Maryland; Forsyth County, North Carolina; Jackson, Mississippi; or northwestern suburbs of Minneapolis, Minnesota).

[b] Model 2: adjusted for Model 1+ marital status (married, widowed, divorced, separated, or never married), and annual household income (<$25,000, $25,000–$49,999, $50,000–$74,999, or ≥$75,000).

[c] middle-to-older adulthood: age 40–50 years; young adulthood: age 30 years.

[d] Low and high neighborhood socioeconomic were defined based on the median neighborhood socioeconomic at each life stage

Note: $P_{interaction}$ based on Model 2

Number of events:

Black participants n=584; White participants: n=1,021

© 2024 Lawrence WR et al. *JAMA Network Open.*

eTable 6. Associations Between Life Epoch Neighborhood Socioeconomic Status and Premature Mortality at Age 70 Cut-Off Overall and Stratified by Sex, Atherosclerosis Risk in Communities Study, 1996-2020

	Overall (10,664)		Men (4,488)		Women (6,176)	
Life Epoch [e]	Model 1 [a] HR (95% CI)	Model 2 [b] HR (95% CI)	Model 1 [c] HR (95% CI)	Model 2 [d] HR (95% CI)	Model 1 [c] HR (95% CI)	Model 2 [d] HR (95% CI)
Middle Adulthood neighborhood SES						
T1 (lowest)	1.51 (1.15-2.00)	1.20 (0.90-1.60)	1.65 (1.12-2.43)	1.27 (0.84-1.90)	1.41 (0.95-2.09)	1.19 (0.78-1.79)
T2	1.34 (1.06-1.69)	1.20 (0.95-1.52)	1.24 (0.90-1.71)	1.10 (0.79-1.53)	1.45 (1.03-2.03)	1.32 (0.94-1.87)
T3 (highest)	Reference	Reference	Reference	Reference	Reference	Reference
P_{trend}	0.003	0.20	0.01	0.26	0.08	0.41
$P_{interaction}$				<0.001		
Young Adulthood neighborhood SES						
T1 (lowest)	1.16 (0.91-1.48)	1.02 (0.79-1.30)	1.25 (0.88-1.77)	1.06 (0.74-1.52)	1.06 (0.75-1.49)	0.97 (0.69-1.37)
T2	0.97 (0.77-1.22)	0.92 (0.73-1.15)	1.26 (0.92-1.72)	1.18 (0.86-1.61)	0.73 (0.52-1.03)	0.70 (0.50-0.98)
T3 (highest)	Reference	Reference	Reference	Reference	Reference	Reference
P_{trend}	0.26	0.94	0.19	0.69	0.79	0.84
$P_{interaction}$				<0.001		
Childhood neighborhood SES						
T1 (lowest)	0.87 (0.66-1.14)	0.85 (0.64-1.11)	0.83 (0.56-1.22)	0.77 (0.52-1.14)	0.91 (0.62-1.32)	0.91 (0.63-1.33)
T2	1.05 (0.83-1.32)	1.06 (0.84-1.34)	1.01 (0.73-1.40)	1.00 (0.72-1.39)	1.09 (0.79-1.51)	1.11 (0.80-1.54)
T3 (highest)	Reference	Reference	Reference	Reference	Reference	Reference
P_{trend}	0.39	0.32	0.40	0.24	0.72	0.74
$P_{interaction}$				<0.001		

Abbreviations: CI, confidence interval; HR, hazard ratio; T, tertile; SES, socioeconomic status.

[a] Model 1: adjusted for age (years; continuous) sex (male or female), and study center (Washington County, Maryland; Forsyth County, North Carolina; Jackson, Mississippi; or northwestern suburbs of Minneapolis, Minnesota).

[b] Model 2: adjusted for Model 1+ marital status (married, widowed, divorced, separated, or never married), racial group (Black participants or White participants), and annual household income (<$25,000, $25,000–$49,999, $50,000–$74,999, or ≥$75,000).

[c] Model 1: adjusted for age (years; continuous), and study center (Washington County, Maryland; Forsyth County, North Carolina; Jackson, Mississippi; or northwestern suburbs of Minneapolis, Minnesota).

© 2024 Lawrence WR et al. *JAMA Network Open.*

[d] Model 2: adjusted for Model 1+ marital status (married, widowed, divorced, separated, or never married), racial group (Black participants or White participants), and annual household income (<$25,000, $25,000–$49,999, $50,000–$74,999, or ≥$75,000).
[e] Middle adulthood: age 40–50 years; young adulthood: age 30 years; childhood: age 10 years.
Note: $P_{interaction}$ based on Model 2
Number of events:
Overall n=632; Men n=316; Women: n=316.

© 2024 Lawrence WR et al. *JAMA Network Open.*

eTable 7. Associations Between Life Epoch Neighborhood Socioeconomic Status and Premature Mortality at Age 70 Cut-Off Stratified by Racial Group, Atherosclerosis Risk in Communities Study, 1996-2020

Life Epoch [c]	Black Participants (n=2,776) Model 1 [a] HR (95% CI)	Black Participants (n=2,776) Model 2 [b] HR (95% CI)	White Participants (n=7,888) Model 1 [a] HR (95% CI)	White Participants (n=7,888) Model 2 [b] HR (95% CI)
Middle Adulthood neighborhood SES				
T1 (low)	1.54 (0.93-2.55)	1.08 (0.64-1.81)	1.40 (0.95-2.07)	1.18 (0.79-1.76)
T2	1.25 (0.72-2.19)	1.01 (0.57-1.79)	1.39 (1.08-1.80)	1.28 (0.99-1.66)
T3 (high)	Reference	Reference	Reference	Reference
P_{trend}	0.06	0.72	0.02	0.18
$P_{interaction}$		0.77		
Young Adulthood neighborhood SES				
T1 (low)	1.36 (0.90-2.05)	1.17 (0.77-1.77)	0.99 (0.71-1.39)	0.88 (0.63-1.24)
T2	1.01 (0.63-1.62)	1.00 (0.62-1.60)	0.98 (0.75-1.28)	0.91 (0.70-1.19)
T3 (high)	Reference	Reference	Reference	Reference
P_{trend}	0.09	0.38	0.92	0.43
$P_{interaction}$		0.41		
Childhood neighborhood SES				
T1 (low)	0.85 (0.59-1.22)	0.84 (0.58-1.21)	0.96 (0.63-1.47)	0.92 (0.60-1.40)
T2	1.05 (0.71-1.53)	1.07 (0.73-1.57)	1.07 (0.80-1.42)	1.08 (0.81-1.44)
T3 (high)	Reference	Reference	Reference	Reference
P_{trend}	0.37	0.34	0.97	0.90
$P_{interaction}$		0.95		

Abbreviations: CI, confidence interval; HR, hazard ratio; T, tertile; SES, socioeconomic status.
[a] Model 1: adjusted for age (years; continuous) sex (male or female), and study center (Washington County, Maryland; Forsyth County, North Carolina; Jackson, Mississippi; or northwestern suburbs of Minneapolis, Minnesota).
[b] Model 2: adjusted for Model 1+ marital status (married, widowed, divorced, separated, or never married), and annual household income (<$25,000, $25,000–$49,999, $50,000–$74,999, or ≥$75,000).
[c] Middle adulthood: age 40–50 years; young adulthood: age 30 years; childhood: age 10 years.
Note: $P_{interaction}$ based on Model 2
Number of events:
Black participants n=254; White participants: n=378.

© 2024 Lawrence WR et al. *JAMA Network Open.*
