# Supplementary material for: Neighborhood Socioeconomic Disadvantage Across the Life Course and Premature Mortality
Source: JAMA Netw Open. Author manuscript; Available in PMC 2024 Aug 19. (PMC11307131; doi:10.1001/jamanetworkopen.2024.26243)
Supplement: Supplement Online Content 2. — Data Sharing Statement [file NIHMS2015532-supplement-Supplement_Online_Content_2_.pdf]

Data Sharing Statement

Lawrence. Neighborhood Socioeconomic Disadvantage Across the Life Course and Premature Mortality. *JAMA Netw Open*. Published August 07, 2024.
doi:10.1001/jamanetworkopen.2024.26243

Data
Data available: Yes
Data types: Deidentified participant data
How to access data: ARIC study data are available through the BioLINCC database https://biolincc.nhlbi.nih.gov/home/
When available: With publication

Supporting Documents
Document types: None

Additional Information
Who can access the data: BioLINCC data are available upon reasonable request.
Types of analyses: BioLINCC data are available for any analyses.
Mechanisms of data availability: BioLINCC data are available following completion of NHLBI Research Materials Distribution Agreement.
